# The effect of video observation on the frequency, type, and accuracy of peer feedback in a long jump learning context

**DOI:** 10.3389/fpsyg.2025.1737033

**Published:** 2025-12-29

**Authors:** Hiba Abdelkafi, Ahmed Ghorbel, Mohamed Yaakoubi, Mohamed Moncef Kammoun, Mohammed Issa Alsaeed, Adnene Gharbi, Omar Trabelsi

**Affiliations:** 1 Department of Education Sciences, The High Institute of Applied Studies in Humanities of Mahdia, University of Monastir, Mahdia, Tunisia; 2 Research Unit: Physical Activity, Sport and Health, UR18JS01, National Observatory of Sport, Tunis, Tunisia; 3 The High Institute of Sport and Physical Education, University of Jendouba, Kef, Tunisia; 4 The High Institute of Sport and Physical Education, University of Sfax, Sfax, Tunisia; 5 Department of Biomechanics & Motor Behavior, College of Sport Science & Physical Activity, King Saud University, Riyadh, Saudi Arabia

**Keywords:** cooperative learning, long jump, multimedia-based learning, sport pedagogy, video-based feedback

## Abstract

**Background:**

Video technology facilitates feedback provision in sport pedagogy, benefiting both teacher/coach and peer feedback. Most studies focus on attributing learning gains to video-based peer feedback, but none examine how the dynamics of feedback itself change to mediate the effects of video observation (VO) on learning outcomes.

**Objectives:**

This study compares the frequency, type, and accuracy of peer feedback based on direct observation (DO) and VO in a long jump learning context.

**Methods:**

Forty-one sports science students (*M*_age_ = 20.13 ± 0.71) participated in a four-session long jump learning unit. Thereafter, they were paired randomly for the experimental procedures: one performed a jump while the other observed. Observers first provided 30 s of verbal feedback based on DO, followed by another 30 s after viewing a video recording of the jump (VO). Roles were then switched. Audio-recordings were transcribed and analyzed for overall feedback frequency. Feedback instances were classified as descriptive or prescriptive, with prescriptive feedback assessed for accuracy. Comparisons were conducted using the Wilcoxon signed-rank test for paired samples.

**Results:**

Video observation significantly increased the overall frequency of feedback (median: 5 vs. 3, mean: 5.3 vs. 2.9, *p* < 0.001) and prescriptive feedback (median: 3 vs. 1, mean: 2.9 vs. 0.7, *p* < 0.001), while descriptive feedback significantly decreased (median: 2 vs. 2, mean: 2 vs. 1.5, *p* = 0.009). Moreover, the accuracy of prescriptive feedback was significantly higher based on VO (median: 100% vs. 50%, mean: 89.5% vs. 38.9%, *p* = 0.002).

**Conclusion:**

These findings help explain the well-documented effects of video-based peer feedback in sport pedagogy. VO empowers learners to engage more socially and focus more on providing prescriptive and accurate feedback, as the video itself provides descriptive information to the performer and a more detailed, controlled observation for the observer.

## 1 Introduction

Motor learning is a highly complex process which involves the integration of sensory inputs, cognitive strategies, and motor execution to achieve individuals’ skilled performance (Wolpert et al., 2011). This adaptive process enables learners to develop and refine skills through guided training and instruction (Magill and Anderson, 2020). Feedback is known as a critical component of motor learning, providing information about performance that helps individuals improve their movement skills.

Contemporary perspectives in motor learning pedagogy recognize feedback as a critical mediator of skill acquisition, with growing emphasis on optimizing both its temporal delivery and qualitative dimensions (Brand et al., 2020; Treschman et al., 2024). Effective feedback requires more than simple error detection. It demands precise alignment between the informational content and the specific developmental needs of the learner (Northcraft et al., 2011), a concept supported by the optimal challenge point framework (Guadagnoli and Lee, 2004). Implementing such tailored feedback in practice poses significant challenges for educators and trainers. These barriers are notably amplified in educational contexts where pedagogues must simultaneously monitor multiple learners (Hodges and Franks, 2002). This has driven a paradigm shift toward participatory learning models.

Peer feedback has emerged as an effective pedagogical tool for skill acquisition, demonstrating remarkable benefits across diverse motor learning contexts (Patterson et al., 2019; Wulf et al., 2010). Recent meta-analytic evidence suggests peer feedback can improve motor performance outcomes compared to no-feedback conditions (Han et al., 2022), underscoring its functional utility.

Traditionally, feedback is delivered by instructors or coaches, but recent pedagogical shifts emphasize the potential of peer feedback as a collaborative, learner-centered strategy (Rink, 2013; Casey and Goodyear, 2015) through Direct Observation (DO). When combined with video technology, peer video feedback becomes a powerful tool that leverages the principles of observational learning. This approach allows learners to pause, review, and objectively analyze performance, facilitating deeper cognitive processing of the movement (Ste-Marie et al., 2012). Research by Ste-Marie et al. (2012) has shown that video observation (VO), particularly when combined with self-modeling techniques, is highly effective for skill acquisition and error detection in sporting activities. The social act of peers discussing these video recordings further enhances understanding by integrating multiple perspectives and creating a collaborative learning environment (Zhu and Carless, 2018). This synergy of cognitive analysis and social interaction makes video-enhanced peer feedback a uniquely effective method for skill development. However, determining the most effective form of peer feedback presents a persistent challenge, particularly in motor learning contexts involving both novice and advanced learners. The decision between feedback types (such as descriptive versus prescriptive) can critically shape the effectiveness of the learning process and the quality of outcomes (Hattie and Timperley, 2007; Wulf and Lewthwaite, 2016).

Descriptive peer feedback in motor learning contexts involves providing objective observations about an athlete’s movements and performance without necessarily suggesting corrections. This type of feedback can be particularly beneficial for more experienced learners who have developed a refined understanding of their sport and can independently analyze their technique to make precise adjustments (Nunes et al., 2020; Rezvani and Yazdi, 2023). However, most studies in this area have focused on teacher-driven descriptive feedback (Rezvani and Yazdi, 2023) or self-assessment contexts (Pasquier et al., 2023), leaving a gap in the understanding of how peers can effectively provide this type of feedback in real-world motor learning settings.

In contrast, prescriptive peer feedback offers specific, actionable advice for performance improvement. This form is generally more suitable for novice learners who may lack the experience to self-diagnose and correct errors effectively (Potdevin et al., 2018; Souissi et al., 2021). Prescriptive feedback provides clear, structured guidance that can accelerate early skill acquisition by reducing the cognitive load associated with error correction (Ericsson and Pool, 2016).

Efforts to improve the feedback process in motor learning have increasingly turned to technology, with VO emerging as a particularly effective approach (Jastrow et al., 2022; Trabelsi et al., 2022). Research consistently shows that VO significantly enhances skill acquisition compared to DO, earning it a strong position in sport pedagogy (Mödinger et al., 2022). In weightlifting, Souissi et al. (2021) highlighted the benefit of using slower playback speeds for feedback, particularly in the context of diagnosing technical errors in complex Olympic lifts. Their research demonstrated that slowing down video replay allows athletes and coaches to more effectively identify and correct specific, rapid technical flaws that are often missed at real-time speed. The advantages of VO also extend to peer feedback, whether descriptive or prescriptive. For example, Palao et al. (2015) showed that VO significantly enhanced hurdle technique learning even when feedback came from peers, while Trabelsi et al. (2020) found that peer feedback based on VO was particularly impactful.

Most prior studies have primarily focused on evaluating the effectiveness of video-based feedback on various dependent variables within motor learning contexts. However, these investigations often overlook the underlying mechanisms and processes of change that may account for the observed beneficial effects. Instead, this study seeks to build on existing evidence by exploring how VO is reshaping the dynamics of peer feedback (specifically its frequency, type, and quality) in motor learning contexts. The choice to examine peer feedback according to these three dimensions (frequency, type, and quality) was intentional. Frequency provides an indicator of learner engagement (Patterson et al., 2019). Type, distinguishing between descriptive and prescriptive feedback, reflects the instructional function of peer comments, with prescriptive feedback generally more impactful for novices (Shute, 2008; Nunes et al., 2020). Finally, quality, operationalized here as the accuracy of prescriptive comments, is crucial since the effectiveness of feedback depends not only on its presence but also on its correctness (Zhou et al., 2021).

Accordingly, this study seeks to examine the effect of VO on the frequency and accuracy of both descriptive and prescriptive peer feedback in the context of long jump learning. Specifically, we hypothesize that: (i) the overall frequency of peer feedback will be significantly higher under the VO condition compared to DO; (ii) this increase will be particularly pronounced for prescriptive feedback; and (iii) the accuracy of prescriptive feedback will also be greater in the VO condition. These hypotheses will be tested within a long jump task involving young adult sports science students, emphasizing the importance of precise movement patterns in this specific motor learning context.

## 2 Methods

### 2.1 Participants

**Figure 1:**
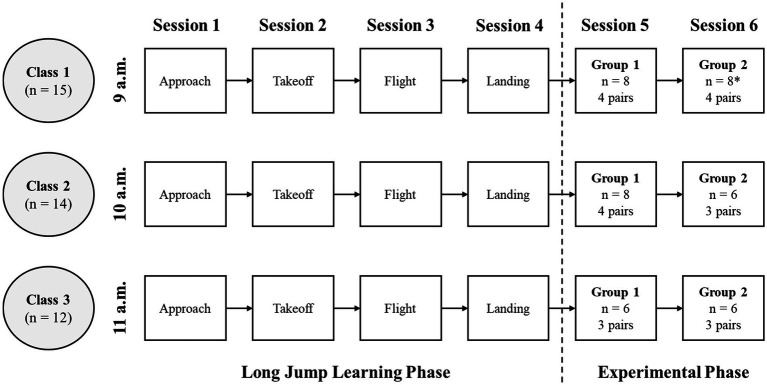
Study flowchart: overview of 60-min learning and experimental sessions. *This group initially consisted of 7 participants, with one additional participant joining from Group 1 to form 4 pairs.

The participants included in this study were 41 first-year sport science students, with a mean age of 20.13 ± 0.71 years, and no dropouts were recorded as all students who met the eligibility criteria and provided consent were included in the study. The group consisted of 17 males and 24 females, all of Tunisian nationality, enrolled at the High Institute of Sport and Physical Education of Kef, Tunisia. They were distributed across three classes: 15 students in the first, 14 in the second, and 12 in the third (Figure 1). For the experimental pairing in Class 1 (*n* = 15), one participant was reassigned during the session to ensure all students worked in pairs, as detailed in the experimental procedure. To be eligible for the study, participants had to meet several criteria. They needed to be in their first year of study to ensure consistency in age and academic level. Furthermore, they were required to have no prior experience in long jump outside formal educational settings, reducing the risk of performance differences due to prior athletic experience. Participants also had to be in good health, with no vision impairments, to prevent bias in the VO component. Finally, those with physical conditions, such as injuries or disabilities that could impact long jump performance, were excluded.

To determine the appropriate sample size for this study, a power analysis was carried out using G*Power software (Faul et al., 2007), focusing on the matched pairs t-test from the t-test family. This analysis, assuming a medium effect size (dz = 0.5), a power of 0.8, and a significance level of *α* = 0.05, indicated that a minimum of 34 participants was necessary, provided the data followed a normal distribution. However, to account for the possibility of non-normal data, a secondary power analysis was performed using the Wilcoxon signed-rank test (matched pairs), which is more appropriate for non-parametric distributions. This approach, based on the minimum achievable relative efficiency (min ARE), recommended a sample size of at least 39 participants under the same power and significance conditions. With 41 participants included in this study, the sample size was therefore considered sufficient to ensure reliable statistical analysis, regardless of the underlying data distribution.

### 2.2 Legal and ethical considerations

This study was conducted in full compliance with all legal and ethical guidelines, consistent with the principles of the Helsinki Declaration. Ethical approval was obtained from the Ethics Committee of the High Institute of Sport and Physical Education of Kef (reference number 0071–2023). All participants provided written informed consent, which included a clear explanation of the study’s objectives, procedures, and potential risks. Furthermore, the necessary administrative permissions were secured from the institution where the study was conducted.

### 2.3 Study procedures

The study was conducted on the long jump sand pit at the athletics field of the High Institute of Sport and Physical Education of Kef, Tunisia. Data collection took place over a six-week period in November and December, with sessions held every Friday during scheduled class hours. Each week, the first group attended at 9 a.m., the second at 10 a.m., and the third at 11 a.m. (Figure 1).

#### 2.3.1 Learning phase

All students took part in four 60-min training sessions focused on long jump (Figure 1), with the primary aim of building the technical understanding necessary for effective peer feedback. Each session addressed one of the four main phases of the long jump: approach, takeoff, flight, and landing. Sessions opened with a 5-min introduction, followed by a 10-min warm-up incorporating both general exercises and targeted movements to activate the muscle groups critical for long jump performance. The core practice segment lasted 40 min and included four structured learning activities, each lasting 9 min, with the final 4 min allocated for cooldown. During the main part of the session, the instructor provided detailed guidance on the correct execution of each phase, highlighting key performance indicators (KPIs) through live demonstrations and video modeling on a tablet. KPIs were derived from established technical models and validated by a panel of three expert long jump coaches. KPIs included technical components for each phase (e.g., consistent stride pattern in approach, full leg extension at takeoff). These criteria provided observers with an objective framework to identify and critique technical execution, shifting feedback from subjective opinion to structured technical analysis. The instructor, a university professor and expert long jump coach, provided explicit, standardized instructions on how to observe and provide constructive feedback. This was defined for participants as feedback that was: (1) Specific, targeting observable techniques (e.g., “your takeoff foot was too far behind the line”); (2) Actionable, providing a clear corrective cue (e.g., “try to plant your foot closer to the board”); and (3) Technical, focusing on biomechanical execution rather than outcome or general effort. These criteria were emphasized and practiced during the learning sessions to prepare students for the subsequent experimental tasks and ensure consistent feedback quality across all participants.” After each demonstration, students practiced the techniques, with opportunities for peer feedback to support their learning.

#### 2.3.2 Experimental phase

To ensure all participating students complete the procedures, two experimental sessions were conducted for each class right after the four learning sessions (Figure 1), as a single 60-min session was insufficient. Each session started with a 10-min warm-up, followed by two practice jump trials per student. Next, the students were paired randomly (Figure 1).

**Figure 2:**
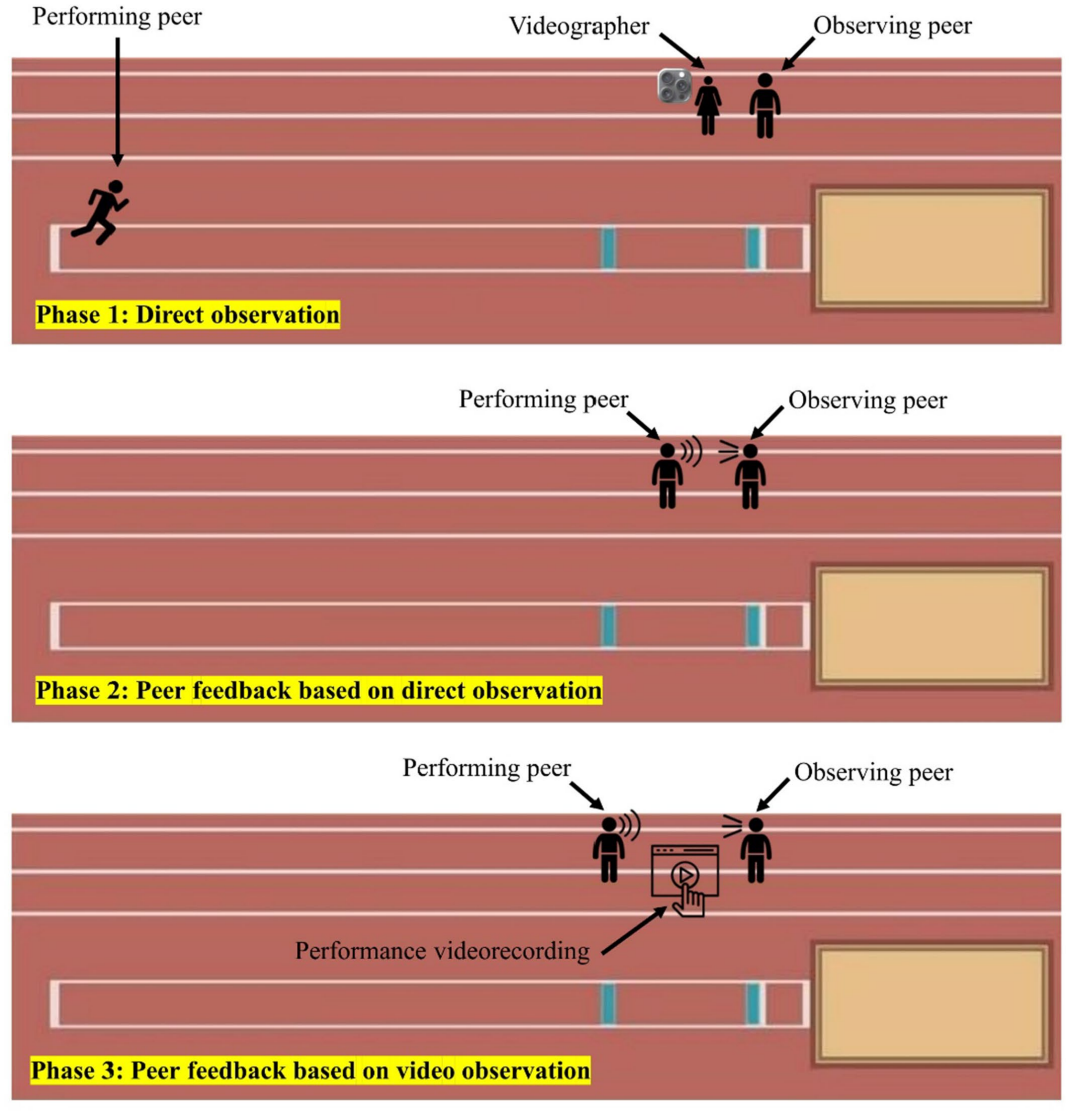
Detailed overview of the experimental session procedures.

In each pair, one student (the performer) executed the jump while the other (the observer) stood slightly behind the takeoff board to observe the jump’s four phases comprehensively (Figure 2). After the jump, the performer rejoined the observer, who then had 30 s to give feedback based on their direct observations.

**Figure 3:**
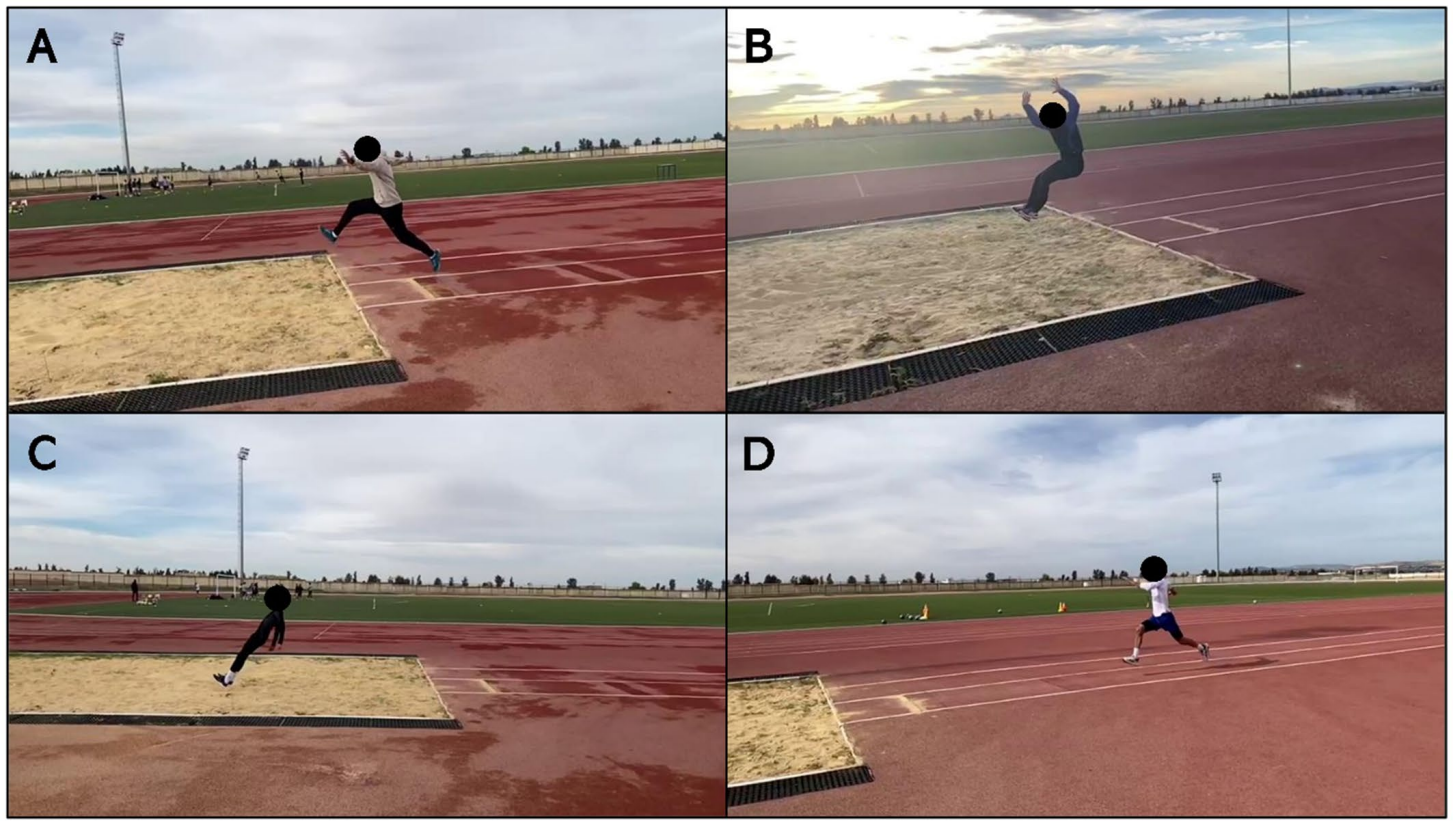
Four screenshots **(A–D)** extracted from video recordings of the long jump performances.

Right after giving their first round of feedback, the observing student was handed a smartphone (Apple Inc. [2022] iPhone 12) showing a high-definition slow-motion recording (1080 p at 120 fps) of the same jump. They could freely interact with the video—replaying, pausing, rewinding, or zooming in to examine specific movements. The video was captured by a researcher stationed near the takeoff area, who filmed the performance in real time using the same device (Figure 2). Sample frames from these recordings are displayed in Figure 3.

After reviewing the video for 90 s, the observer had an additional 30 s to offer feedback, now informed by their video analysis. The students then swapped roles: the observer became the jumper, while the performer took on the observer role for the next trial. To ensure that differences between conditions were attributable solely to the observation modality, the same observer evaluated the same performance in both the DO and VO conditions. Using different performances across conditions would have introduced uncontrolled variability, as each jump contains distinct strengths and technical errors. Keeping the performance constant allowed a direct comparison between real-time observation and video-based review.

During experimental trials, only one pair of students performed at a time while the other participants waited at the opposite end of the field. This arrangement minimized performance anxiety for the active pair, which was essential to ensure that the quantity and quality of feedback provided were based on the observer’s perception of the performance itself, rather than being influenced by the performer’s heightened stress or self-consciousness. When the second student in the performing pair started receiving feedback, the next scheduled pair was called to begin their warm-up routine. This secondary warm-up served to reactivate their muscles and maintain proper preparation levels, counteracting any cooling effects from the initial warm-up period before their turn to perform.

### 2.4 Data collection

All verbal feedback provided after both direct and video observations was systematically audio recorded for analysis. To ensure high-fidelity audio capture, a research member of the team was positioned adjacent to each participant during feedback delivery, maintaining a smartphone microphone (Xiaomi Redmi Note 12, 2023 model) in close proximity to the speaker’s mouth. This secondary recording device was selected for its demonstrated capability to capture vocalizations with exceptional clarity and precision. Following completion of all feedback sessions, the collected audio files were systematically transferred to a Lenovo IdeaPad 5i (2023) laptop for subsequent processing. The final stage of data preparation involved complete verbatim transcription of all recordings using Microsoft Word, with particular attention to preserving the authentic Tunisian dialect (a distinct variant of Arabic) used by participants throughout their feedback.

### 2.5 Data classification

Two qualified members of the research team, possessing specialized expertise in feedback evaluation, conducted a systematic content analysis of the transcribed data. Their primary objective was to quantify all feedback instances generated within the exact 30-s response window following both: (1) direct observation and (2) video observation periods. This quantification was performed without categorical distinction between different feedback types. To ensure analytical rigor, inter-rater reliability was calculated, yielding a strong Cohen’s kappa coefficient of *κ* = 0.87, indicating excellent agreement between coders.

#### 2.5.1 Descriptive and prescriptive feedback classification

The transcripts were then analyzed to categorize feedback instances into descriptive and prescriptive types. For example, one transcript excerpt stated: “*Very good! Your takeoff was perfect, with your toes very close to the line. To improve further, you need to increase your approach speed. Also, try engaging your legs more during takeoff to gain additional distance*.” In this example, the statement “*Your takeoff was perfect, with your toes very close to the line*” represents descriptive feedback as it objectively describes a specific aspect of the performance. The subsequent suggestions, “*you need to increase your approach speed”* and *“try engaging your legs more during takeoff*,” constitute prescriptive feedback as they provide specific technical recommendations for improvement. The initial exclamation “Very good!” was not categorized as either descriptive or prescriptive feedback since it served only as general encouragement without technical content. It was categorized as ‘Other,’ which included general encouragement or off-task comments without instructional content. This approach allowed for clear differentiation between feedback that simply described performance characteristics (descriptive) and feedback that offered direct technical corrections (prescriptive), with each type being counted separately in the analysis. The categorization process maintained this distinction throughout the examination of all transcripts, ensuring consistent classification of feedback instances based on their instructional content and purpose.

#### 2.5.2 Prescriptive feedback accuracy

The final analysis phase evaluated the accuracy of prescriptive feedback provided by students in both observation conditions (direct vs. video-assisted). Feedback accuracy was validated by two researchers and two independent expert long jump coaches (not the instructor), a method aligned with established practice using subject matter experts to ensure unbiased valid assessment (Ericsson et al., 2006). Their consensus determined ground truth for scoring. Accuracy was quantified as the percentage of correct prescriptive suggestions relative to the total prescriptive feedback given for each performance. For instance, if a student offered two prescriptive feedback instances during direct observation (e.g., “*Extend your arms higher during flight*” [accurate] and “*Shorten your approach run*” [inaccurate]), the accuracy index would be 50%. During video review of the same jump, if they provided four prescriptive feedback instances with three being accurate (e.g., “*Delay your arm drop until landing*” [accurate], “Drive your knee higher at takeoff” [accurate], and “*Keep your head lifted during flight*” [accurate]), their accuracy would improve to 75%. An example of inaccurate prescriptive feedback would be advising “*Correct your backward lean at takeoff*” when video analysis confirmed proper forward torso positioning.

The classification and accuracy assessment processes were conducted independently by researchers over 5 months, with periodic reliability checks. Comparative analyses revealed exceptional inter-rater agreement, with Cohen’s kappa coefficients consistently ranging between 0.88 and 0.96, indicating near-perfect consensus in evaluations. To prevent expectation-based bias, the two researchers who coded the feedback and the independent long jump experts who assessed prescriptive-feedback accuracy were blinded to the observation condition (DO vs. VO), as transcripts and feedback excerpts were presented without any identifiers.

### 2.6 Data analysis

Data collation yielded 8 clean datasets, forming four paired observations: (1) overall feedback frequency (DO vs. VO), (2) descriptive feedback instances (DO vs. VO), (3) prescriptive feedback instances (DO vs. VO), and (4) accuracy percentage of explicit feedback (DO vs. VO). Normality of differences for each of these dependent pairs was checked using the Shapiro–Wilk test and was rejected for all at *p* < 0.05. Therefore, the non-parametric Wilcoxon signed-rank test was used to assess differences in distributions and compare medians (whether the median difference is significantly different from zero) instead of comparing means in a parametric approach.

Effect size was reported using *r*, which was calculated as follows: $\;r=\frac{z}{\sqrt{n}}$ . The larger the value of 𝑟, the stronger the effect or difference between the paired samples. *r* ranges from 0 to 1, with values above 0.5 indicating a large effect size (Cohen, 2013). All statistical analyses were conducted using IBM SPSS Statistics software, version 29 (IBM Corp., 2022).

## 3 Results

### 3.1 Descriptive statistics

Table 1 presents the descriptive statistics for feedback provision frequency, comparing the number of feedback instances based on DO and VO. Overall feedback instances showed a median of 3.0 (IQR = 2.0) for DO and 5.0 (IQR = 2.0) for VO, with means of 2.90 ± 0.92 and 5.30 ± 1.45, respectively. Descriptive feedback had a median of 2.0 (IQR = 1.5) for DO and 2.0 (IQR = 1.0) for VO, with means of 2.00 ± 0.78 and 1.49 ± 0.59, respectively. For prescriptive feedback, DO had a median of 1.0 (IQR = 1.0), and VO had a higher median of 3.0 (IQR = 2.0), with means of 0.66 ± 0.73 and 2.93 ± 1.13, respectively. Other types of feedback showed a median of 0.0 (IQR = 1.0) for DO and 1.0 (IQR = 1.0) for VO, with means of 0.39 ± 0.54 and 0.83 ± 0.70, respectively.

**Table 1:** Descriptive statistics of feedback provision frequency (number of feedback instances).

Feedback type	Condition	Med.	IQR	*M*	SD	Min	Max
Overall feedback	DO	3.0	2.0	2.90	0.92	2	5
	VO	5.0	2.0	5.30	1.45	3	8
Descriptive feedback	DO	2.0	1.5	2.00	0.78	1	4
	VO	2.0	1.0	1.49	0.59	1	3
Prescriptive feedback	DO	1.0	1.0	0.66	0.73	0	2
	VO	3.0	2.0	2.93	1.13	1	5
Other types	DO	0.0	1.0	0.39	0.54	0	2
	VO	1.0	1.0	0.83	0.70	0	3

M, mean; SD, standard deviation; Med., median; IQR, interquartile range; DO, direct observation; VO, video observation.

### 3.2 Inferential statistics

As shown in Table 2, the Wilcoxon test revealed a significant difference in overall feedback instances in favor of VO (*Z* = −5.598, *p* < 0.001), with a large effect size (*r* = 0.87). For descriptive feedback, the difference between DO and VO was also significant (*Z* = 2.660, *p* = 0.009), but with a medium effect size (*r* = 0.42), and interestingly, in favor of DO. For prescriptive feedback, the test revealed a significant difference in favor of VO (*Z* = −5.518, *p* < 0.001), with a large effect size (*r* = 0.86). To sum up, the statistical analyses showed a significant increase in overall and prescriptive feedback instances from DO to VO, both with large effect sizes. In contrast, there was a significant decrease in descriptive feedback from DO to VO, with a medium effect size.

**Table 2:** Comparison of overall, descriptive, and prescriptive feedback instances based on direct vs. video observation.

Type	Condition	NR (MR)	PR (MR)	Ties	Wilcoxon test			
					Z	*p*	*r*	Effect size
Overall feedback	DO vs. VO (*n* = 41)	1^a^ (2.5)	38^b^ (20.5)	2^c^	−5.598	<0.001	0.87	Large
Descriptive feedback	DO vs. VO (*n* = 41)	17^a^ (8.00)	4^b^ (8.00)	20^c^	2.660	0.009	0.42	Medium
Prescriptive feedback	DO vs. VO (*n* = 41)	0^a^ (0.0)	39^b^ (19.5)	2^c^	−5.518	<0.001	0.86	Large

DO**, d**irect **o**bservation; VO**, v**ideo **o**bservation; NR**, n**egative **r**anks; PR**, p**ositive **r**anks; MR**, m**ean **r**ank; ^a^VO < DO; ^b^VO > DO; ^c^VO = DO.

Table 3 presents the descriptive and inferential statistics for prescriptive feedback accuracy percentages. For DO, the median accuracy was 50% (IQR = 62.5), and the mean accuracy was 38.89% ± 40.42%. For VO, the median was 100% (IQR = 25), and the mean was 89.54% ± 20.63%. The Wilcoxon test revealed a significant difference in prescriptive feedback accuracy between DO and VO (*Z* = −3.081, *p* = 0.002), with a large effect size (*r* = 0.73). Notably, this analysis was limited to 18 participants, as 23 provided no prescriptive feedback instances following DO.

**Table 3:** Comparison of prescriptive feedback accuracy percentages based on direct vs. video observation.

Condition	Med.	IQR	*M*	SD	NR (MR)	PR (MR)	Ties	Wilcoxon test			
								Z	*p*	*r*	Effect size
DO	50	62.5	38.89	40.42	2^a^ (9.5)	13^b^ (14.9)	3^c^	−3.081	0.002	0.73	Large
VO	100	25	89.54	20.63							

DO, direct observation; VO, video observation; Med., median; IQR, interquartile range; M, mean; SD, standard deviation; NR, negative ranks; PR, positive ranks; MR, mean rank; ^a^VO < DO; ^b^VO > DO; ^c^VO = DO.

This analysis included data from only 18 participants due to 23 participants providing 0 prescriptive feedback instances after DO.

## 4 Discussion

The present study evaluated the differential effects of observation modality (direct vs. video-based) on frequency and accuracy aspects of peer feedback in a motor learning context, specifically focusing on long jump performance. The primary aim was to examine how VO influences the quantity and quality of peer feedback compared to DO. Consistent with our hypotheses, the results demonstrate that VO significantly enhanced the feedback process. Specifically, it led to a greater overall frequency of feedback, a pronounced increase in the provision of prescriptive feedback, and improved accuracy of those prescriptive suggestions compared to DO. The analysis revealed several noteworthy outcomes, particularly regarding differences in overall feedback output, the proportion of descriptive versus prescriptive feedback between the two observation conditions, and differences in the accuracy of prescriptive feedback in DO compared to VO.

### 4.1 Interpretation of key findings

The findings indicate that VO generated a significantly higher volume of peer feedback than DO. Crucially, this advantage cannot be attributed to a longer feedback delivery time, as both conditions were limited to the same strict 30-s window. Instead, it indicates that the additional video observation period enhanced learners’ cognitive preparation, enabling them to formulate and articulate higher-quality feedback within an identical time constraint. This observation aligns with findings from video-based peer feedback research (Trabelsi et al., 2020) and underscores the role of technology in fostering collaborative learning dynamics. Specifically, participants noted that VO facilitated active interaction, critical reflection, and discussion. This demonstrates how the technology served as a common reference point for collaborative analysis. By enabling learners to review details repeatedly, VO enhanced memory retention, a critical factor in motor learning were precise, context-specific feedback drives skill refinement.

The collaborative learning environment reported in VO groups (Trabelsi et al., 2020) suggests video tools may foster feedback literacy-learners’ ability to understand, process, and apply feedback (Carless and Boud, 2018). When sports students repeatedly analyze videos together, they develop: (1) judgment skills to distinguish critical from trivial errors, (2) language precision to articulate technical corrections, and (3) receptivity to receiving critiques. This tripartite development mirrors the ‘peer feedback maturation’ model observed in writing education (Nicol, 2021), implying VO’s benefits may extend beyond motor learning to broader educational competencies.

This advantage extends beyond mere quantity of feedback. Cognitive and confidence-related mechanisms also explain the results. VO’s structured review process likely boosted observers’ self-efficacy (Deshpande et al., 2023), reducing hesitation in delivering prescriptive feedback. When learners could verify their observations through repeated video analysis, they grew more confident in their critiques, mitigating the fear of embarrassment linked to incorrect feedback (Ajabshir, 2014). In contrast, DO’s ephemeral nature appeared to heighten uncertainty, prompting participants to default to vague or descriptive feedback to avoid judgment—a tendency corroborated by Patterson et al. (2019), who emphasize that effective peer feedback requires observational skills systematically enhanced by VO.

These findings align with and extend Schmidt’s Schema Theory by demonstrating how VO enhances both the *recall* and *recognition* schemas critical for motor learning. The video’s capacity to provide permanent, reviewable performance records strengthens the reference of correctness (recall schema), while repeated error detection builds the recognition schema needed for in-movement adjustments (Schmidt, 1975). Notably, VO’s prescriptive feedback may accelerate schema formation by explicitly linking errors to corrective actions (e.g., “*knee drive too low*” → “*extend 15° higher*”), a process less accessible in DO’s real-time constraints. This explicitness mirrors the ‘guided discovery’ approach (Wulf, 2007), a pedagogical strategy that balances learner exploration with structured guidance. In this framework, targeted external feedback optimizes learning efficiency by helping learners focus their attention on the most relevant information (e.g., key performance indicators), thereby reducing cognitive load and preventing practice errors from becoming ingrained. This accelerates the discovery of correct movement solutions compared to unguided trial-and-error.

These cognitive benefits, however, must be contextualized within participants’ beginner-level proficiency. While VO’s advantages are clear, its effectiveness may hinge on how well learners’ observational skills—deliberately developed during the initial training sessions—can be applied when using video technology. Marques and Corrêa (2016) caution that purely descriptive feedback (even via video) may not suffice for novices to independently correct errors. Thus, VO’s pedagogical value might be maximized when paired with structured guidance to direct attention to critical performance elements. This aligns with principles of observational learning, where the efficacy of video feedback is enhanced by expert modeling (Ste-Marie et al., 2012) or tools like checklists that cue learners to key features of the movement.

Crucially, the benefits of VO extend to feedback quality. The data demonstrated substantial enhancements in the accuracy rates of prescriptive feedback when participants utilized VO compared to DO. This shift in feedback quality aligns with prior research suggesting that video-based tools uniquely support motor skill acquisition in complex tasks like the long jump, where analyzing multiple movement phases is essential (Souissi et al., 2021; Rohbanfard and Proteau, 2013). The controllability of VO—allowing learners to pause, zoom, and rewind—likely reduced cognitive load, enabling deeper processing of kinematic details (e.g., foot placement, knee drive) and more precise corrections. This mechanistic advantage is further supported by cognitive load theory (Ouwehand et al., 2025), which posits that reducing extraneous load (e.g., through slow-motion replay) frees cognitive resources for error detection and prescriptive problem-solving.

Notably, the decline in descriptive feedback under VO suggests learners prioritized actionable corrections over observational statements when video tools were available. By providing actionable information about how to adjust movement patterns, prescriptive feedback enabled learners to modify key motor skill components, thereby enhancing performance. This mirrors findings by Fadde and Sullivan (2013), where video review improved learners’ ability to identify *how* to fix errors rather than merely describing them. For example, in baseball, players using video feedback generated 40% more actionable corrections than those relying on DO. Similarly, in the long jump, VO’s detailed playback allowed learners to isolate and articulate specific technical errors (Souissi et al., 2024), such as improper takeoff angle or landing posture. Ultimately, VO’s capacity to capture nuanced performance aspects fosters substantive peer discussions and aligns with frameworks emphasizing extrinsic feedback for motor learning optimization (Marques and Corrêa, 2016).

### 4.2 Implications for practice

This study’s findings offer several important practical applications for educators, coaches, and curriculum designers working in motor skill development. The robust evidence supporting VO’s effectiveness in generating high-quality prescriptive feedback suggests traditional DO approaches may need reconsideration, particularly for complex skills like the long jump where precise technical feedback is crucial. For practitioners, the key implication is the need to incorporate structured VO protocols into training sessions.

Research demonstrates that when students use frameworks for analysis (e.g., checklists identifying key technical components), their ability to generate meaningful prescriptive feedback improves dramatically (Fadde and Sullivan, 2013). Coaches should develop phase-specific worksheets (approach, takeoff, flight, and landing) with prompts guiding error identification and correction. VO offers significant time efficiency advantages. In settings with high instructor-to-learner ratios, VO enables detailed feedback without constant expert oversight, effectively extending “instructional bandwidth” (Palao et al., 2015).

Learners can review performances asynchronously while structured peer sessions maximize engagement. An important benefit is VO’s role in developing metacognitive skills. Repeated video analysis cultivates “observational expertise” – the ability to critically assess movement patterns (Schmidt and Lee, 2025). This transfers beyond immediate contexts, equipping students with analytical frameworks for future skill acquisition.

Educators should view VO as both a feedback tool and means to foster independent learning. Institutions must invest in technology infrastructure and professional development. While smartphones provide basic video capabilities, optimal implementation benefits from dedicated devices with slow-motion/zoom functions and secure sharing platforms. Crucially, coaches need training in designing video analysis tasks, facilitating peer feedback, and scaffolding learner independence.

For curriculum design, VO activities should progress from highly structured (beginner) to open-ended (advanced). Beginners benefit from clear performance criteria and modeling, while advanced learners can handle more independent analysis. Future implementations could explore combining VO with motion capture or augmented reality for enhanced feedback. VO should transition from occasional tool to central pedagogical component, offering benefits for both short-term performance and long-term skill development.

### 4.3 Limitations of the study

While this study provides valuable insights into video observation’s advantages for peer feedback, several limitations must be acknowledged. First, the single-measurement design precludes analysis of how feedback quality evolves with repeated practice sessions. Although pragmatically necessary, this constraint means we cannot determine whether VO’s benefits are maintained or enhanced over extended training periods. Future research should employ longitudinal designs to track feedback development across multiple learning sessions.

Second, our participant pool-consisting exclusively of young adult university students-raises questions about generalizability. The cognitive maturity and educational background of this demographic may not reflect how younger learners or those with different educational experiences would benefit from VO. Subsequent studies should examine these effects across diverse age groups and skill levels to establish broader applicability.

Methodologically, while expert evaluation of video recordings provided a reasonable standard for assessing feedback accuracy, this approach carries inherent subjectivity. Incorporating objective biomechanical measures (e.g., force plate data, 3D motion capture) in future work could yield more precise and quantifiable assessments of both performance and feedback validity, particularly for complex skills like the long jump, though that feedback needs to be simplified for ease of understanding and appropriate for the learning phase.

The peer feedback paradigm itself presents additional considerations. Despite random pairing, social dynamics and interpersonal relationships may have influenced feedback quality and honesty. Anonymous feedback systems or blended peer-expert models could help mitigate these potential biases in subsequent investigations.

An additional limitation concerns potential familiarity effects. First, the fixed order of conditions (DO always preceding VO) may have allowed students to refine their approach. Second, within the DO condition itself, a ‘second-speaker effect’ was possible, where the second student in a pair could have benefited from observing their partner’s feedback first. However, the large effect sizes observed for VO, along with converging evidence from prior counterbalanced studies, suggest that its superiority is unlikely to be explained by order or practice alone. Nevertheless, future research should employ a fully counterbalanced design and analyze within-pair order effects to definitively rule out these potential familiarity influences.

Furthermore, a key methodological consideration is the structure of the observation periods. In the DO condition, participants provided feedback immediately after a single, real-time observation. The VO condition, by contrast, provided an additional 90-s period for repeated and detailed video review before feedback was given. While this structure is ecologically valid and represents a core benefit of using video technology, it introduces a potential confound: the observed advantages of VO may be attributable to this opportunity for repeated observation and analysis rather than the video modality itself. Future studies could investigate this distinction by comparing a single viewing of a video replay against the repeated review allowed here.

Most significantly, the study’s focus on immediate outcomes leaves unanswered questions about long-term skill retention. Motor learning theory emphasizes the critical distinction between short-term performance gains and enduring skill acquisition (Schmidt and Wrisberg, 2008). Future research should incorporate delayed retention tests to determine whether VO’s advantages in feedback quality translate to sustained improvements in movement execution and competition performance, particularly for skills requiring high levels of automatization like the long jump (Wulf, 2013).

## 5 Conclusion

While this paper primarily focuses on how VO is transforming peer feedback dynamics (including frequency, type, and quality), future studies should continue exploring this promising area. The findings of this study suggest that VO, as compared to DO, significantly enhanced learners’ ability to provide verbal feedback on their peers’ long jump performances. This improvement was particularly pronounced in prescriptive feedback (characterized by specific suggestions for performance enhancement) while the level of descriptive feedback, which focuses on recounting observed actions without evaluative judgment, remained relatively stable across both conditions. Furthermore, the accuracy of prescriptive feedback was markedly higher in the VO condition. These results highlight the pedagogical value of VO in motor learning, as it promotes deeper reflective analysis and enables learners to offer more detailed, precise, and actionable feedback. This creates a stronger foundation for peer-supported learning, potentially leading to more effective skill development.

## Data Availability

The raw data supporting the conclusions of this article will be made available by the authors, without undue reservation.
